# The significance of proline and glutamate on butanol chaotropic stress in *Bacillus subtilis* 168

**DOI:** 10.1186/s13068-017-0811-3

**Published:** 2017-05-11

**Authors:** Gumpanat Mahipant, Atchara Paemanee, Sittiruk Roytrakul, Junichi Kato, Alisa S. Vangnai

**Affiliations:** 1 Biological Sciences Program, Faculty of Science, Chulalongkorn University, Bangkok, 10330 Thailand; 2 Department of Biochemistry, Faculty of Science, Chulalongkorn University, Bangkok, 10330 Thailand; 3 Proteomics Research Laboratory, Genome Institute Biotechnology, National Center for Genetic Engineering and Biotechnology (BIOTEC), Pathum Thani, 12120 Thailand; 4 Department of Molecular Biotechnology, Graduate School of Advanced Sciences of Matter, Hiroshima University, Hiroshima, 739-8530 Japan; 5 Center of Excellence on Hazardous Substance Management (HSM), Chulalongkorn University, Bangkok, 10330 Thailand

**Keywords:** Butanol tolerance, *Bacillus subtilis*, Proteomics, Chaotropicity, Proline

## Abstract

**Background:**

Butanol is an intensively used industrial solvent and an attractive alternative biofuel, but the bioproduction suffers from its high toxicity. Among the native butanol producers and heterologous butanol-producing hosts, *Bacillus subtilis* 168 exhibited relatively higher butanol tolerance. Nevertheless, organic solvent tolerance mechanisms in Bacilli and Gram-positive bacteria have relatively less information. Thus, this study aimed to elucidate butanol stress responses that may involve in unique tolerance of *B. subtilis* 168 to butanol and other alcohol biocommodities.

**Results:**

Using comparative proteomics approach and molecular analysis of butanol-challenged *B. subtilis* 168, 108 butanol-responsive proteins were revealed, and classified into seven groups according to their biological functions. While parts of them may be similar to the proteins reportedly involved in solvent stress response in other Gram-positive bacteria, significant role of proline in the proline–glutamate–arginine metabolism was substantiated. Detection of intracellular proline and glutamate accumulation, as well as glutamate transient conversion during butanol exposure confirmed their necessity, especially proline, for cellular butanol tolerance. Disruption of the particular genes in proline biosynthesis pathways clarified the essential role of the anabolic ProB-ProA-ProI system over the osmoadaptive ProH-ProA-ProJ system for cellular protection in response to butanol exposure. Molecular modifications to increase gene dosage for proline biosynthesis as well as for glutamate acquisition enhanced butanol tolerance of *B. subtilis* 168 up to 1.8% (vol/vol) under the conditions tested.

**Conclusion:**

This work revealed the important role of proline as an effective compatible solute that is required to protect cells against butanol chaotropic effect and to maintain cellular functions in *B. subtilis* 168 during butanol exposure. Nevertheless, the accumulation of intracellular proline against butanol stress required a metabolic conversion of glutamate through the specific biosynthetic ProB-ProA-ProI route. Thus, exogenous addition of glutamate, but not proline, enhanced butanol tolerance. These findings serve as a practical knowledge to enhance *B. subtilis* 168 butanol tolerance, and demonstrate means to engineer the bacterial host to promote higher butanol/alcohol tolerance of *B. subtilis* 168 for the production of butanol and other alcohol biocommodities.

**Electronic supplementary material:**

The online version of this article (doi:10.1186/s13068-017-0811-3) contains supplementary material, which is available to authorized users.

## Background

*n*-Butanol (referred to as butanol hereafter) has been intensively used as an important organic solvent (used interchangeably with solvent hereafter) in chemical industries, and recently becomes an attractive renewable alternative biofuel over traditional biofuels due to its higher energy content and greater physical and chemical properties. Due to fossil resource shortage, butanol has been increasingly produced generally by a conventional process using anaerobic bacteria of the genus *Clostridium* [1]. However, butanol fermentation in Clostridia normally suffers from several phenotypic problems, including complication of acidogenesis-to-solventogenesis metabolic shift, by-product formation, and most importantly, butanol toxicity to the producing cells, which results in microbial cell damage and low butanol productivity [2]. Alternatively, butanol production can also be achieved using heterologous bacterial hosts with metabolically engineered butanol synthetic pathway, such as *Escherichia coli*, *Pseudomonas putida*, *Lactobacillus brevis*, and *Bacillus subtilis* [3–5]. These bacteria share common traits as industrial relevant strains, such as high growth rate and genetic competency; however, one of the most desirable and requisite host characteristics for alcohol bioproduction is microbial tolerance to the alcohol product [6, 7]. Previous studies on microbial alcohol tolerance indicated that among common facultative anaerobic host organisms used for biofuel and alcohol production, *B. subtilis* not only exhibits the highest butanol tolerance (up to 2%, vol/vol) [4], but also has high potential use as a bioproduction platform for various alcohols including butanol, and other biocommodities [8, 9]. Despite superior butanol tolerance of *B. subtilis*, organic solvent tolerance mechanisms have been mostly studied in Gram-negative bacteria, while relatively less information has been described in either *B. subtilis* or Gram-positive bacteria [10].

Up to present, studies on Gram-positive cellular chemical stress responses that may impart tolerance to solvents including alcohols have been mainly conducted with *Bacilli*, *Clostridia*, and *Actinobacteria*, and so far reveal the following proposed mechanisms, some of which are similar to those of Gram-negative bacteria [1] and yeast [11]: (i) general stress responses involving molecular chaperones and sigma factor B (SigB or σ^B^)-dependent activities as reported in *Bacilli* exposed to ethanol or *iso*-propanol; (ii) changes in cell morphology and sporulation; (iii) cell membrane adaptation and cell surface modification; (iv) efflux pumps; and (v) metabolic detoxification [10]. Nevertheless, bacterial solvent tolerance and adaptation are not contributed by an individual mechanism, but are considered complex multigenic traits that require the integrated functions and systematic changes of genes and proteins, of which the information can be acquired by omics approaches [7, 10, 12]. While global and general stress response mechanisms in Gram-positive bacteria have been extensively studied by omics-based analysis [13–15], comprehensive study on tolerance and adaptation towards solvents as well as alcohol, especially butanol, is limited [16].

Consequently, to fill in the knowledge gap in understanding the molecular responses and tolerance of *B. subtilis* toward butanol, this research applied a comparative proteomics approach to analyze proteins expressed differentially in *B. subtilis* strain 168 in a normal growth condition and in response to butanol stress. Proteomics data combined with the available genomic information of *B. subtilis* 168 provided effective techniques to elucidate gene function at the protein level. Further analysis of this study then revealed the first evidence of the deficiently explored role of compatible solutes, proline, and glutamate, in butanol tolerance of *B. subtilis*, despite the fact that this protective role has been previously and mainly reported in the osmotic-stressed microbial cells, partly in ethanol-stressed yeast [17], and ethanol-stressed *P. putida* [18]. Expression of genes related to the synthesis and metabolic network of proline and glutamate as well as molecular studies of the related mutants substantiated the involvement of these compatible solutes as one of the butanol stress response and tolerance mechanisms in *B. subtilis*. These findings provide insight into the biochemical basis of butanol stress responses in *B. subtilis* and will be beneficial as a means to engineer the bacterial host to express specific enzymes, thereby conferring higher butanol tolerance of the host for the production of butanol and other alcohol biocommodities.

## Results

### Butanol tolerance of *B. subtilis* 168

**Fig. 1:**
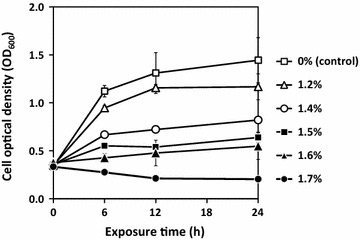
Butanol tolerance of *B. subtilis* 168. Cells were inoculated in LB medium and exposed to butanol at various concentrations (vol/vol). Cell growth was measured to evaluate butanol tolerance in comparison to that of a non-butanol exposed cell control. Data are means of the results from at least three individual experiments. *Error bars* indicate standard errors

Butanol tolerance has been reported in several bacteria including *B. subtilis*; however, there are large variations of the test outcomes because of the use of different types of tolerance assays and test conditions [5, 6, 19, 20]. In general, there are two main assays to assess bacterial cell tolerance toward alcohol: cell growth assay and survival assay of high-density cells [7]. For batch and fed-batch fermentation, one of the important traits for a chemical-producing host is a good cell growth under the stress of alcohol product [7, 21]. Consequently, a growth-based butanol tolerance assay was used in this study, and butanol tolerance was monitored by optical density measurement. Using this assay, the cells prepared for tolerance tests at various butanol concentrations in this study were in early exponential phase so that their inherent responses to butanol were reflected accurately, and they were at low density to minimize genetic heterogeneity within the population [7]. In addition, chemical tolerance capability of cells is influenced primarily by the medium composition. Accordingly, to measure their full response to toxic chemicals, cells are typically tested for tolerance in a complex, rich medium, such as LB medium or Terrific broth, in which nutrients are sufficient to promote energy-intensive cellular activities such as efflux pump, chaperone activities, cell damage repair [7], as well as other protective mechanisms, if any. In this study, *B. subtilis* 168 cells were tested with various concentrations of butanol under the indicated test conditions. Butanol tolerance was also evaluated by measuring the ability of the cell to grow; thus tolerance was determined by the bacterial specific growth rate. The specific growth rate of the non-butanol exposed cells was 0.234 ± 0.020 h^−1^, which decreased to 0.188 ± 0.002, 0.156 ± 0.001, and 0.109 ± 0.003 h^−1^ when cells were exposed to butanol at 1.2, 1.4, and 1.6%, respectively, while cells exposed to 1.7% butanol did not grow (Fig. 1). Although previous studies reported that the butanol tolerance of *B. subtilis* ranged from 1 to 2.25% (vol/vol) butanol under the indicated test conditions [19, 22], in this study *B. subtilis* 168 apparently tolerated butanol concentration up to 1.6% (vol/vol) because of differences in the test conditions, including the inoculum age, the initial cell density, and the monitoring method.

### Proteomics analysis of *B. subtilis* 168 under butanol stress

**Fig. 2:**
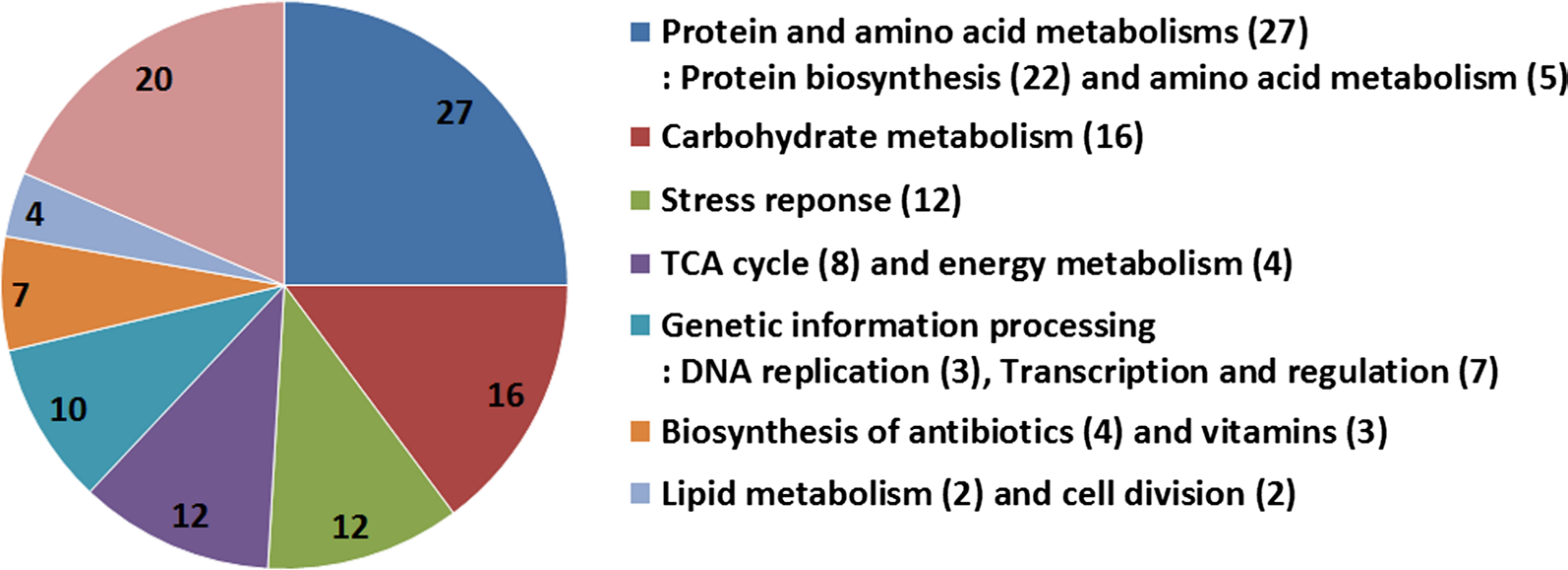
Distribution of butanol-responsive proteins categorized according to their biological functions from proteomics analysis of *B. subtilis* 168 exposed to 1.4% (vol/vol) butanol under the indicated test conditions

To elucidate the response mechanism(s) of *B. subtilis* 168 to butanol stress at the protein level, a comparative proteomics analysis was conducted. In general, proteomics studies of bacteria are analytically restricted by low protein abundances [23]. In this case, overcoming this problem was challenging because the greater the butanol concentration applied to *B. subtilis* 168 cells, the more stressed the cells became, which resulted in a lower growth rate and a lower cell biomass (Fig. 1). Consequently, cells subjected to butanol stress at 1.2 and 1.4% butanol concentration, which enabled them to maintain reasonable growth rates, were selected for further proteomics analysis. A differential analysis of proteins extracted from cells exposed to butanol for 6 h was conducted, and the results were compared to those extracted from non-butanol exposed cells (i.e., non-stressed, LB-grown cells). Using a one-dimensional SDS-PAGE protein separation (Additional file 1), followed by in-gel tryptic digestion, and LC–MS/MS analysis, 2230 peptides were identified initially. Further analysis identified 108 butanol-responsive proteins with ≥1.5-fold change in expression, of which 104 were upregulated and four were downregulated. Subsequently, the proteins were categorized into seven groups according to their biological functions (Fig. 2; Additional file 2) including (in parenthesis, the total number of proteins in each group; the number of upregulated proteins, and the number of downregulated proteins): protein and amino acid metabolism (27; 24, 3) [consisting of protein biosynthesis (22; 19, 3) and amino acid metabolism (5; 5, 0)], carbohydrate metabolism (16; 16, 0), stress response (12; 12, 0), tricarboxylic acid (TCA) cycle and energy metabolism (4; 4, 0), genetic information processing (10; 10, 0), biosynthesis of antibiotic and vitamin (7; 7, 0), lipid metabolism and cell division (9; 9, 0), and others (20; 19, 1).

**Fig. 3:**
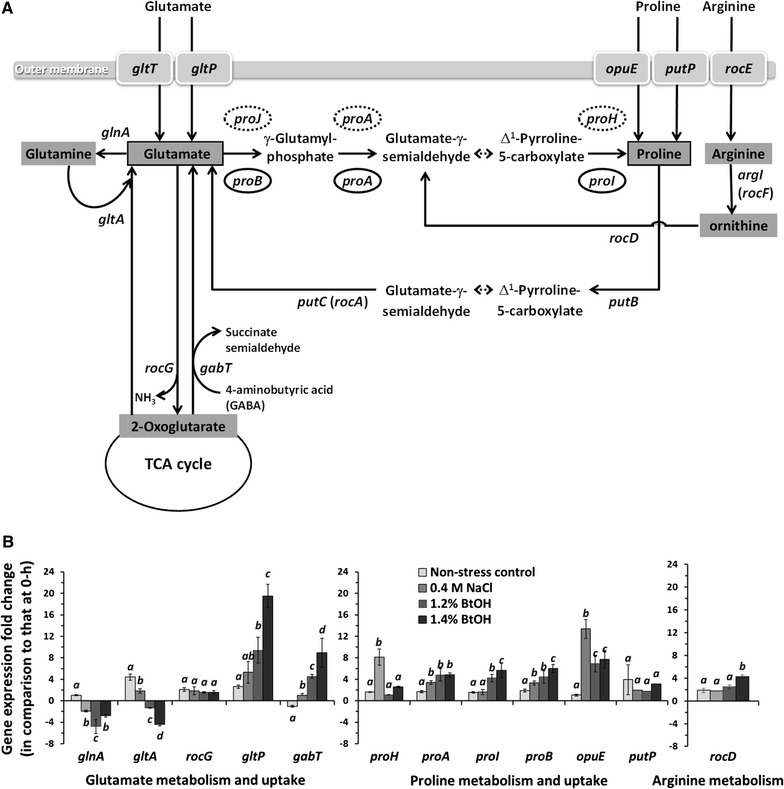
Metabolic network of glutamate, proline, and arginine metabolism in *B. subtilis* 168 (**A**). Expression of genes involved in glutamate, proline, arginine metabolism, and protein transporter (**B**). The numbers are expressed as fold change of gene expression from cells treated with 1.2–1.4% butanol for 1 h in comparison to that of cells prior to the treatment. The results are the mean of at least three independent treatments. The *italic letters* indicate significant difference from the non-treatment control value of each gene (*p* < 0.05)

For the most part, the identified proteins engaged in the butanol stress response of *B. subtilis* 168 are similar to previously reported proteins involved in solvent stress response in Gram-positive bacteria [10]. A thorough analysis showed the upregulation of a SigB-positive regulator (RsbRA) and six SigB-controlled gene products in response to butanol stress (Additional file 2) including ribonuclease R (*rnr* gene product) and a general stress protein 20U (*dps* gene product), each of which were previously reported to directly involve in ethanol stress response in *B. subtilis* [24]. In addition, the analysis prominently revealed the significant upregulation of Δ^1^-pyrroline-5-carboxylate dehydrogenase (RocA or PutC; EC 1.2.1.88) by 1.7- to 1.9-fold, and ornithine-oxo-acid transaminase (RocD; EC 2.6.1.13) by 1.9- to 2.1-fold (Fig. 3A; Additional file 2). Interestingly, this result showed that although the cells had access to excessive and available nutrients in the LB medium, they responded to butanol stress by upregulating enzymes related to proline and glutamate metabolism. Accordingly, since the involvement of proline as well as glutamate toward butanol stress response in *B. subtilis* 168 was deduced; thus, it became the focus for further investigation.

### Involvement of proline and glutamate in the *B. subtilis* 168 response to butanol stress: expression levels of genes involved in glutamate and proline metabolisms, the intracellular accumulation of glutamate and proline, and the effects of the exogenous addition of these amino acids

Proline and glutamate are well-reported bacterial-compatible solutes, which are synthesized de novo or taken up from the surrounding medium into cells for cellular protection from heat and salt stresses [25, 26]. In this study, the proteomics analysis inferred their involvement in the butanol stress response of *B. subtilis* 168; therefore the expression of genes involved in their metabolic networks, i.e., synthesis, degradation, and uptake systems, were determined quantitatively to assess the dynamic cellular response to butanol stress. In addition, because their role as osmoprotectants has been established in the bacterial salt stress response, a gene expression analysis of cells grown in a moderate salinity environment was conducted as a control for comparison purposes. For this analysis, to minimize the expression of genes involved in the uptake of other nutrients in the surrounding environment, cells were cultured in a chemically defined SMM (without exogenous addition of proline and glutamate), and they were treated for 1 h with 1.2 and 1.4% butanol, as well as 0.4 M NaCl [27], prior to total RNA extraction for a quantitative reverse transcription (RT) PCR analysis. Under these conditions, expression of particular genes would truly reflect the cellular response to butanol stress.

In *B. subtilis* 168, glutamate is taken up from the surrounding environment by the main glutamate–aspartate symporter (GltT) or a proton–glutamate symporter (GltP), while it is synthesized predominantly by a reductive amination of 2-oxoglutarate catalyzed by glutamate synthase (GltA) [28], or through 4-aminobutyric acid (GABA) metabolism by 4-aminobutyrate aminotransferase (GabT) [29], and through catabolic route of proline by Δ^1^-pyrroline-5-carboxylate dehydrogenase (PutC or RocA) [30] (Fig. 3A). Glutamate degradation occurs through the reaction catalyzed by glutamate dehydrogenase (RocG) and glutamate synthetase (GlnA) (Fig. 3A). Upon butanol stress, the glutamate synthesis gene *gabT* and glutamate transporter gene *gltP* were strongly upregulated up to 9 ± 3- and 20 ± 2-fold, respectively (Fig. 3B), while GltT was not upregulated (data not shown). Although previous reports showed that *gltP* upregulation and GltP activity vary depending upon the type of carbon source and stage of cell growth [31], its role in cellular stress protection was not assessed [13]. Our findings here clearly indicated its involvement in glutamate uptake in butanol-stressed cells (Fig. 3B). Further analysis also showed that *gltA*, and glutamate degradation gene, *glnA*, were downregulated approximately five and threefold, respectively, to minimize glutamate consumption via other pathways (Fig. 3B). The downregulation of *gltA*, which is normally categorized in glutamate synthesis route from glutamine, probably occurred as a regulatory reflect caused by the decrease of the intracellular glutamine level [32]. The overall gene expression results substantially pointed out that *B. subtilis* 168 increases its intracellular glutamate level upon butanol exposure.

A similar experiment was conducted simultaneously to examine the proline metabolism of *B. subtilis* 168. Proline is generally taken into cells via a specific transporter, and it can be produced from the precursor glutamate, through either anabolic or osmoadaptive biosynthesis routes [30]. A gene expression analysis revealed that an osmotic- or ethanol–stress-inducible transporter gene [33], *opuE*, was highly upregulated by 13 ± 2- or 6 ± 2-fold under salt stress or butanol stress, respectively (Fig. 3B), while the expression level of a high-affinity proline transporter gene, *putP*, existing as part of the *putBCP* operon [30], remained unchanged (Fig. 3B). On the contrary, if proline was exogenously provided under the non-stressed condition (2 mM in SMM), the *putP* expression level was enhanced by 8.0 ± 4.2-fold indicating that proline effectively induces the catabolic *putBCP* operon. This result agrees with the fact that PutP is primarily utilized for scavenging of proline as a nutrient for cell growth through glutamate, but reportedly not for stress-protective purposes [30]. In addition, the expression of genes that encode enzymes in the proline anabolic biosynthetic route, including γ-glutamyl kinase (ProB), γ-glutamyl phosphate reductase (ProA), and Δ^1^-pyrroline-5-carboxylate reductase (ProI) increased significantly upon butanol stress (6 ± 2-, 5 ± 0.5-, and 6 ± 1-fold, respectively), while expression level of the *proHJ* operon encoding the isozymes ProI and ProB, respectively, was not altered statistically (Fig. 3B). Furthermore, the analysis showed a fourfold increase in the expression level of *rocD* gene-encoding ornithine aminotransferase in arginine catabolism, as this gene is commonly induced by the presence of intracellular proline, ornithine, and arginine [34]. The results strongly indicated that *B. subtilis* 168 requires the acquisition of proline to protect cells against butanol stress. Our findings indicated that the expression of *proBA* transcript, but not the *proHJ* transcript, was induced sensitively by butanol stress. A further analysis was required (as described subsequently) to determine the role of each enzyme system whether the anabolic ProB-ProA-ProI system or the osmoadaptive ProH-ProA-ProJ system acts as a preferred proline biosynthesis route from glutamate in response to butanol exposure.

**Fig. 4:**
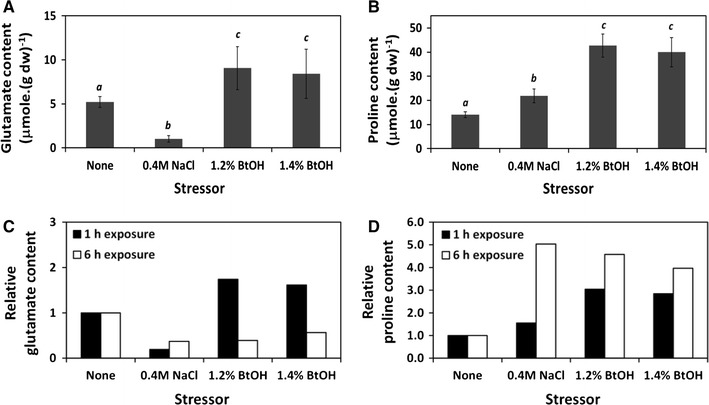
Intracellular glutamate and proline in *B. subtilis* 168 challenged with salt or butanol in comparison to those in the non-stressed cell control (indicated as “*None*”). Content of intracellular glutamate (**A**) and proline (**B**) measured when cells were treated with each stressor for 1 h. Relative values of intracellular glutamate content (**C**) and intracellular proline content (**D**) determined at 1 and 6 h of cell exposure to each stressor. The *italic letters* indicate significance level (*p* < 0.05)

Because the quantitative analysis of gene expression of butanol-stressed cells indicated a requirement for proline and glutamate, the accumulation of intracellular proline and glutamate upon butanol stress was examined. The contents of these two amino acids were determined from cells cultivated in a minimal medium and exposed to butanol for 1 and 6 h, while those of the normal cells and cells stressed by moderate salinity were used as controls. Upon 1 h of butanol stress at 1.2 and 1.4% butanol concentrations, the glutamate and proline contents in *B. subtilis* 168 increased rapidly by two and threefold, respectively, from their basal levels, i.e., from 5 ± 1 and 14 ± 1 μmol (g dry cell weight)^−1^ to approximately 9 ± 3 (Fig. 4A) and 43 ± 5 μmol (g dry cell weight)^−1^ (Fig. 4B), respectively. Following a 6 h prolonged exposure of cells to butanol and salt stress, a significant increase (up to fivefold) of the intracellular proline pool was observed compared with that of the non-stressed cells (Fig. 4D), while the glutamate pool decreased by approximately 2.5-fold (Fig. 4C). As this result agrees with the phenomenon previously observed in *Bacilli* subjected to an osmotic upshift with 0.4 M NaCl [27], a conclusion could be made that the tolerance of *B. subtilis* 168 toward butanol is associated predominantly with the substantial increase in the pool of intracellular proline, which acts as an effective compatible solute in response to butanol stress, whereas glutamate is accumulated initially and is utilized subsequently as a precursor for proline biosynthesis.

**Fig. 5:**
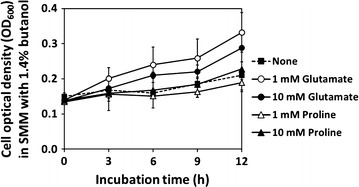
Effect of exogenous addition of glutamate and proline on a growth-dependent butanol tolerance of *B. subtilis* 168. Cells were grown in SMM supplemented with 1.4% (vol/vol) butanol. Cell optical density was interval monitored to represent cell butanol tolerance. As a control, cells were grown in normal conditions without butanol addition (referred to as “*None*”) or in the presence of glutamate at 1 or 10 mM, or proline at 1 or 10 mM, each of which was exogenously added in the medium. Data are means of the results from at least three individual experiments. *Error bars* indicate standard errors

As our results accentuated the roles of intracellular glutamate and proline in the cellular response and tolerance to butanol stress, the investigation was then conducted whether cells can alleviate butanol stress by importing these two amino acids from the surrounding medium. A growth-dependent butanol tolerance assay was conducted in SMM in the absence or presence of either 1 or 10 mM glutamate and proline. Then, cell tolerance to 1.4% butanol was monitored as cell growth by cell optical density measurements. The exogenous addition of glutamate at both concentrations markedly enhanced butanol tolerance as the cell biomass was increased twofold after 12 h of incubation, and the cell doubling time decreased by 2.5-fold, as shown that a 27 h doubling time of cells grown without glutamate was down to approximately 11 h (Fig. 5). However, the exogenous addition of proline did not increase butanol tolerance (Fig. 5). Furthermore, because proline is enzymatically converted from arginine, the effect of an external addition of arginine, at 1 and 10 mM, on butanol tolerance was also examined. The exogenous arginine failed to increase the butanol tolerance of *B. subtilis* 168 (data not shown). The overall results indicated that glutamate, but not proline, was taken up from the surrounding environment as a part of cellular butanol stress response (Fig. 5), and that it was converted subsequently to proline (Fig. 4) for cellular butanol stress protection.

### Physiological function of the proline biosynthesis route ProB-ProA-ProI and the GltP transporter associated with the butanol stress response in *B. subtilis* 168

**Fig. 6:**
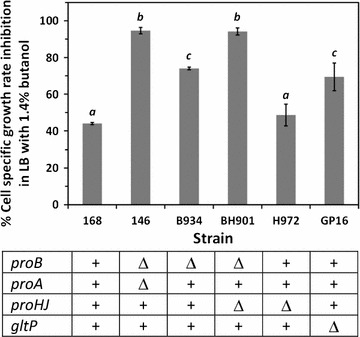
Specific growth-rate inhibition percentage of *B. subtilis* wild-type and the mutant strains with 1.4% (vol/vol) butanol exposure. The* symbols* represent genotype of the wild-type and each mutant strain: +, Δ represent the presence and absence of the indicated gene, respectively. During growth in LB medium and butanol exposure, cell optical density was interval measured to determine percentage of cellular growth inhibition. Data are means of the results from at least three individual experiments. *Error bars* indicate standard errors. The *letters* indicate significance level (*p* < 0.05)

Even though the butanol stress-mediated gene expression results implied the involvement of particular biological systems, i.e., the ProBA-ProI-proline biosynthesis system, and the GltP-glutamate transporter, in cellular butanol stress defenses (Fig. 3B), it was essential to elucidate their physiological functions under butanol-stressed conditions. An analysis was conducted with four proline auxotrophic mutants of *B. subtilis* 168 (genetic descriptions in parentheses and Table 1): strain 146 (168Δ*proBA*), strain B934 (168Δ*proB*), strain BH901 (168Δ*proBHJ*), and strain H972 (168Δ*proHJ*), and a glutamate transporter disrupted mutant, strain GP16 (168Δ*gltP*) (each strain is referred to by its number hereafter). The test was performed when cells were grown in the presence of 1.4% butanol in LB medium where sufficient nutrients were available for other cellular mechanisms that are required for cell protection. Despite slight specific growth rate differences, the first four mutants showed the expected proline auxotrophic phenotype as they did not grow significantly without proline supplementation in SMM, while they grew normally in LB medium as well as in SMM with 2 mM proline (i.e., non-stress condition) (Additional file 3). All the mutants were then subjected to a growth-dependent butanol tolerance test in LB medium containing 1.4% (vol/vol) butanol. The specific growth rates of cells were monitored, and they were compared to their specific growth rates in the absence of butanol, and expressed as a specific growth-rate inhibition percentage. Compared among all strains, the strain with higher growth-rate inhibition percentage represented the strain with higher sensitivity to butanol. The wild-type strain 168 showed moderate butanol tolerance, as its specific growth rate in the presence of butanol was one-half of that of its growth rate in the absence of butanol (Fig. 6). When *proBA* or *proB*-*proHJ* was completely disrupted in strains 146 and BH901, respectively, cells were highly susceptible to butanol stress as the specific growth rate was inhibited by 95% (Fig. 6). In contrast, when *proHJ* was disrupted alone in strain H972, butanol tolerance did not differ from that of the wild-type strain. These results strengthened our previous finding that *B. subtilis* 168 triggered and used a distinctive gene set encoding the ProBA-ProI system as the predominant proline biosynthesis route against butanol stress, even in nutrient-sufficient conditions. Nevertheless, when tested with strain B934, in which *proB* was disrupted, but *proHJA* was intact, cells grew slowly under butanol stress with an approximately 70% specific growth-rate inhibition (Fig. 6). In this case, these results may imply that a well-known osmoadaptive proline production system, ProHJA, is somehow triggered in the *proB*-disrupted mutant to cope with butanol stress. In addition, although several glutamate transporters are known in *B. subtilis* 168 [35], the partially inhibited specific growth rate of GP16 mutant in the butanol tolerance test highlighted the important role of GltP in the glutamate uptake for cellular protection from butanol stress.

**Table 1:** Bacterial strains used in this study

Strains	Relevant genotype and characteristic(s)	Source	Remark^a^
168	A wild-type *Bacillus subtilis* (*trpC2*)	BGSC^b^	BGSC 1A1
146	168*pro*(*AB*)^−^ A proline auxotroph strain with γ-glutamyl phosphate reductase gene (*proA*) and γ-glutamyl kinase gene (*proB*) disruption	BGSC^b^	BGSC 1A652
GP16	168∆(*gltP*::*cam* ^*r*^) A strain with glutamate transporter gene disruption	This study	BMGC 162
B934	168∆(*proB*::*spc* ^*r*^) A proline auxotroph strain with γ-glutamyl kinase gene disruption	This study	BMGC 163
BH901	168∆(*proB*::*spc* ^*r*^) ∆(*proHJ*::*cam* ^*r*^) or B934∆(*proHJ*::*cam* ^*r*^) A proline auxotroph strain with *proB*, ∆^1^-pyrroline-5-carboxylate reductase gene (*proH*) and a *proB*-like γ-glutamyl kinase gene (*proJ*) disruption	This study	BMGC 164
H972	168∆(*proHJ::cam* ^*r*^) A proline auxotroph strain with ∆^1^-pyrroline-5-carboxylate reductase gene (*proH*) and a *proB*-like γ-glutamyl kinase gene (*proJ*) disruption	This study	BMGC 161
HK	168 carrying pHK vector	This study	BMGC 165
HK-GPOX	168 carrying pHK-gltPOX A recombinant strain with an overexpressed GltP under P43 promoter	This study	BMGC 167
HK′-HJOX	168 carrying pHK-HJOX A recombinant strain with an overexpressed ProHJ under its original promoter	This study	BMGC 166

*cam* ^*r*^ chloramphenicol-resistant gene, *spc* ^*r*^ spectinomycin-resistant gene

^a^The recombinant strains generated in this study were deposited at BIOTEC Culture Collection, National Center for Genetic Engineering and Biotechnology, Pathum Thani, Thailand (shown as BMGC number)

^b^BGSC: Bacillus Genetic Stock Center (Columbus, OH, USA)

### Overexpression of proteins involved in the proline biosynthesis route and glutamate uptake to enhance butanol tolerance in *B. subtilis* 168

**Fig. 7:**
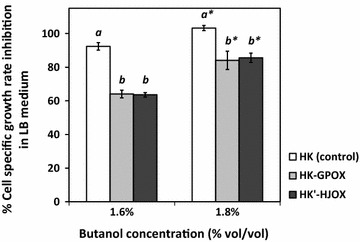
Specific growth-rate inhibition percentage of *B. subtilis* wild-type and the recombinant strains with the overexpressed *gltp* or *proHJ* gene. Each recombinant *B. subtilis* strain with the overexpressed *gltP* (HK-GPOX) and with the overexpressed *proHJ* (HK′-HJOX) was challenged with 1.6 and 1.8% butanol. Cell optical density was interval measured to determine the percentage of specific growth inhibition in comparison to that of the recombinant *B. subtilis* strain harboring the pHK empty vector (a control). Data are means of the results from at least three individual experiments. *Error bars* indicate standard errors. The *italic letters* indicate significance level among strains at each butanol concentration (*p* < 0.05)

Previous reports stated that high levels of intracellular proline as a compatible solute, acquired through a cellular uptake system and the osmotic-inducible ProHJ-ProI biosynthesis route, is required for cell survival under osmotic stress [36]. Subsequent efforts to increase the osmotolerance of cells were achieved by supplying another proline biosynthesis gene set, i.e., *proBA*-*proI* for intracellular proline overproduction. Nevertheless, ProB is typically regulated through feedback inhibition; therefore, a number of studies modified *proB* and *proBA* to relieve this effect and to enhance intracellular proline production and accumulation [37]. In the present study, proline was also shown to play a role in cellular protection against butanol stress. By adopting the same concept to increase intracellular proline production, the addition of another proline biosynthesis gene set, *proHJ*, which is not subject to feedback inhibition control, as well as a gene-encoding glutamate uptake system, *gltP*, were overexpressed to enhance butanol tolerance of *B. subtilis* 168. Each gene was cloned into the pHK vector (Table 1) under the control of the constitutive P_43_ promoter [19], and the resulting plasmids were transformed into *B. subtilis* 168. A recombinant strain, HK, harboring an empty pHK vector was also generated and used as a control in all tests. The transcript level of *gltP* in the HK-GPOX recombinant strain (Table 1) increased by 170-fold compared with that of the control (Additional file 4). In contrast, the overexpression of *proHJ* under P_43_ promoter in a recombinant strain was unsuccessfully generated, probably because of a severe, adverse effect on cell growth. Alternatively, a recombinant strain with a higher *proHJ* gene dosage was successfully generated with its intact promoter in the pHK′ vector, namely HK′-HJOX (Table 1). The quantitative analysis revealed that the *proHJ* transcript level increased by 372-fold (Additional file 4). The specific growth rate of the HK and HK-GPOX strains in LB medium was comparable at approximately 0.123 ± 0.002 h^−1^, while that of HK′-HJOX was slightly slower (0.100 ± 0.002 h^−1^) (Additional file 3). This adverse impact on cell growth was probably caused by the overloaded intracellular proline generated from *proHJ* overexpression and partly by antibiotic stress under the selective conditions. Each recombinant strain was subjected subsequently to a growth-dependent butanol tolerance test in LB medium containing 1.6 and 1.8% (vol/vol) butanol, and the specific growth-rate inhibition percentage was then determined. Similar to that of the wild-type strain, the HK control did not tolerate butanol at both concentrations (Fig. 7). When ProHJ or GltP was overexpressed, the HK′-HJOX and HK-GPOX strains showed less susceptibility to butanol stress as the specific growth-rate inhibition was approximately at 60 and 80% when tested with 1.6 and 1.8% butanol, respectively (Fig. 7). These results indicated that increasing the expression of enzymes that are responsible for the second proline biosynthesis route (ProHJ), or a glutamate-transporting protein (GltP) considerably enhanced butanol tolerance in *B. subtilis* 168.

## Discussion

Butanol is highly toxic to bacterial cells not only because of its hydrophobicity, but also because it causes a chaotropic effect that reduces water activity and severely destabilizes cellular macromolecules [38]. Thus, these detrimental effects have been considered to be critical hurdles that restrict microbial biocatalyst performance during fermentative or non-fermentative butanol production [2]. The key determinant for more efficient butanol production in natural butanol producers, such as *Clostridia*, and alternative butanol-producing hosts is to ameliorate butanol sensitivity or enhance cellular butanol tolerance [5]. Because *B. subtilis* has been reported to be one of the promising hosts with a remarkably higher butanol tolerance compared with other commonly used butanol-producing hosts [4], understanding its butanol stress response that may in part involve in butanol tolerance could be a key improvement of a robust host for butanol synthetic production, and, thus, it was the focus of this study.

Despite the differences of the organisms, genetic traits, and the experimental conditions, the omics-based analyses of butanol-treated *B. subtilis* revealed the typical responses of genes, and their corresponding proteins, similar to other solvent-tolerant bacterial strains including those in SigB-dependent stress regulon [31, 39, 40] as well as proteins involved in stabilizing protein structure, lipid metabolism, and membrane composition modification to maintain cellular integrity [38, 41–46]. Nevertheless, it is worthwhile to note some differences. While a native butanol-producing strain, *Clostridium acetobutylicum*, responded to butanol-challenged conditions with 102 responsive proteins that are mainly involved in amino acid metabolism and protein synthesis in addition to solvent formation-related proteins [43], other potential heterologous hosts for butanol production exhibited various numbers of differentially expressed proteins (generally at 1.5- to 2-fold changes) when subjected to butanol stress, the majority of which are transporters, oxidative stress response proteins, and proteins related to energy metabolism [41–46]; these include (in brackets, the numbers of butanol-responsive proteins assessed by proteomic analysis of each organism) *E. coli* [997] [44], *P. putida* [138] [41], *Staphylococcus warneri* SG1 [108] [42], *Saccharomyces cerevisiae* [>300] [47], and *Synechocystis* sp. PCC 6803 [177] [45]. In this study, among 108 differentially expressed proteins in butanol-treated *B. subtilis*, the role of glutamate- and proline-associated solvent tolerance was uncovered. Although it has been reported previously that glutamate and/or proline are among several compatible solutes that are produced to counteract or mitigate osmotic, acid, heat, and chaotropic stresses in several bacteria including *B*. *subtilis* [48], their acquisition or entry point to biosynthetic routes, as well as their diverse roles in cellular stress protection have not yet been reported in the butanol stress response. Previous reports showed that *B. subtilis* directly acquires exogenously provided glycine betaine and proline using a specific bacterial transporter system for cellular thermoprotection [25], while it relies on glycine betaine that is mainly synthesized from choline, a biosynthetic precursor taken up from the surrounding environment for high-osmolarity stress [49]. For the butanol stress response in *B. subtilis* 168, our analysis indicated that proline is in fact an effective compatible solute and that its intracellular accumulation is required to protect against the chaotropic effect of butanol and to maintain cellular functions. However, coping with butanol stress or enhancing the butanol tolerance of *B. subtilis* 168 could not be achieved simply by the exogenous addition of proline. That is because while the proline transporter PutP is used strictly for the acquisition of proline to serve as carbon and nitrogen sources, another transporter OpuE, which is upregulated likely as a result of the induction of the salt/butanol stress-induced SigB-responsive *opuE* P-2 promoter [33], was reported to exhibit saturated kinetics and a moderate proline transport capacity [50]. On the other hand, it was clearly shown here that the cellular defense against butanol stress in *B. subtilis* 168 relied on proline that was synthesized from glutamate that was taken up from the growth medium through a specific glutamate transporter, GltP, and which accumulated during the initial period of butanol exposure, and was transiently converted to proline. Accordingly, it was apparently shown that exogenous addition of glutamate could stimulate cell growth in the presence of butanol and conferred butanol tolerance in *B. subtilis* 168.

Proline biosynthesis from glutamate in *B. subtilis* 168 generally proceeds through two routes, i.e., anabolic proline biosynthesis by the ProB-ProA-ProI system in which ProB is subjected to feedback inhibition in a proline-sufficient growth medium, or the osmotically induced ProH-ProA-ProJ system [13]. Interestingly, the disruption of the *proB* or *proBA* genes, but not *proHJ*, strongly impaired the butanol tolerance of *B. subtilis* 168, as shown by the higher cellular-specific growth-rate inhibition of the corresponding mutants, compared with the wild-type strains in LB medium containing 1.4% butanol. This finding revealed the important role of the ProB-ProA-ProI route of proline biosynthesis in response to butanol stress even when cells were grown in a nutrient-rich medium. Previous reports have shown that *B. subtilis* 168 responds to high salinity (1 M NaCl) by increasing its intracellular proline content up to 350 μmol (g dry cell weight)^−1^ [27]. In such a case, because ProB in the ProB-ProA-ProI proline synthetic route is regulated by feedback inhibition, this pathway has been considered to be an unsuitable route for proving cells with large amount of proline for osmotic stress protection [51]. In contrast, it was shown in this study that a lower proline content was required for the butanol chaotropic stress response in *B. subtilis* 168 as the intracellular proline concentration was approximately 50 μmol (g dry cell weight)^−1^ under the stress test conditions. Moreover, it has been reported that most aliphatic alcohol chaotropic stressors such as ethanol and butanol at concentrations lower than 25 and 7.7% (w/v), respectively, cause chaotropicity, but do not trigger turgor changes or generate cellular osmotic stress [38, 48]. As a result, despite the fact that cellular osmotic stress was used as a control condition for comparisons in this study, it was unraveled that *B. subtilis* 168 clearly required the ProB-ProA-ProI proline biosynthetic pathway for proline acquisition as a part of the butanol chaotropic stress response mechanism. In addition to the expanding knowledge described here of the role of the ProB-ProA-ProI proline biosynthetic pathway in the butanol stress response in *B. subtilis* 168, increasing the tolerance of this promising butanol-producing host to 1.8% butanol was achieved by overexpressing the second proline biosynthesis route (ProHJ) or a specific glutamate-transporting protein (GltP).

## Conclusions

In summary, *B. subtilis* 168 exhibited relatively high butanol tolerance by expressing several butanol stress responsive proteins involving in a variety of complicated responsive mechanisms. The results from this study indicate a significant role of proline in glutamate–proline–arginine metabolism as one of the key cellular responses during butanol exposure. The importance of the ProB-ProA-ProI proline biosynthetic pathway, which is generally known as an anabolic route, was emphasized as the cellular response to butanol chaotropicity. Exogenous addition of glutamate as a precursor for further metabolic conversion to proline demonstrated a potential approach to enhance butanol tolerance in *B. subtilis* 168. The candidate proteins revealed by proteomics analysis in this study including those in glutamate–proline metabolism require further studies and will be beneficial for rational engineering of a more robust butanol-producing host.

## Methods

### Bacterial strains, growth conditions, and a butanol tolerance test

The bacterial strains used in this study are listed in Table 1. *B. subtilis* 168 was routinely grown in agar-solidified Luria–Bertani (LB) or in LB liquid medium at 37 °C with shaking at 200 rpm. Butanol tolerance test was conducted as a growth-dependent assay [7] in two types of medium either in: (i) the rich undefined LB medium [19] or (ii) Spizizen minimal medium (SMM) consisting of (g L^−1^): 2 (NH_4_)_2_SO_4_, 14 K_2_HPO_4_, 6 KH_2_PO_4_, 1 sodium citrate, 0.25 MgSO_4_, 5 glucose, and trace elements (mg L^−1^): 5.5 CaCl_2_, 15.3 FeCl_3_·6H_2_O, 1 MnCl_2_·4H_2_O, 1.7 ZnCl_2_, 0.43 CuCl_2_·2H_2_O, 0.6 CoCl_2_·6H_2_O, 0.6 NaMoO_4_·2H_2_O, and 0.47 Na_2_SeO_4_, l-tryptophan, l-phenylalanine [52]. Cells (2%, vol/vol, inoculum) were grown to reach an early exponential phase with an optical density (OD_600_) of 0.15–0.3 before butanol was added to the final concentration ranging from 0 to 2% (always expressed as vol/vol). Cell turbidity, measured as OD_600_, was determined at 6 h interval up to 24 h of butanol exposure, while cell colony counting was also occasionally performed to confirm cell survival. Cell tolerance to butanol at various concentrations was evaluated at 6 h butanol exposure by cell-specific growth rate (h^−1^), which was calculated by: ln (*Nt*_*6*_/*Nt*_0_)/6, where *N* is the number of cells at time *t*, or the specific growth-rate inhibition percentage, which was calculated by: $100 \times \frac{{\mu {\text{C}} - \mu {\text{B}}}}{{\mu {\text{C}}}},$ where *µ*B is a specific growth rate of cells under butanol stress condition, and *µ*C is a specific growth rate of a cell control under a normal growth condition [53].

A growth-dependent butanol tolerance with glutamate or proline or arginine supplement (at final concentration of 1 and 10 mM) was conducted in SMM containing 1.4% butanol. Cell turbidity was then measured at time interval.

### Comparative proteomics analysis of the normal and butanol-treated *B. subtilis* 168

#### Protein preparation and separation

Cells were cultured in 10 ml LB medium as described above, and treated with butanol at 1.2 and 1.4% (vol/vol). After 6 h exposure, the treated cells were collected, washed with Tris–EDTA (TE) buffer twice, re-suspended in sodium dodecyl sulfate (SDS) solution (0.5%, wt/vol), and disrupted on ice using a sonicator with an MS73 ultrasonic homogenizer microtip probe at a 40% power scale (Bandelin Sonopuls HD2200, Germany) (3 cycles of 3 min sonication time and 1 min pulse). The cell lysate was centrifuged at 15,000 ×*g* for 3 min, and the supernatant was kept. Protein concentration was determined using a modified Lowry method with bovine serum albumin as a standard protein. Fifteen micrograms of protein was separated on 12.5% SDS-PAGE and stained with Coomassie Brilliant Blue G-250.

#### Protein analysis, identification, and quantification

The gel was horizontally sliced into eight rows, in-gel digested with trypsin, and analyzed via GeLC-MS/MS technique [Proteomics Laboratory, Genome Institute, National Center for Genetic Engineering and Biotechnology (BIOTEC), Thailand] [54]. DeCyder MS differential analysis software (DeCyder MS, GE Healthcare) was used for protein quantitation. Protein search and identification was conducted using MASCOT (version 2.2 Matrix Science, London, UK) with the following parameters: NCBI as a non-redundant database; *Bacillus subtilis* as a taxonomy focus; enzyme trypsin; 3-missed cleavages allowed; carbamidomethylation as a fixed modification; methionine oxidation as a variable modification; peptide tolerance-1.2 Da; MS/MS fragment ion tolerance-0.6 Da. The biological function was assigned to the identified proteins according to the Uniprot or KEGG databases. Proteins matched by the detection of at least two unique peptides per protein, or with Mascot protein matching score of more than 5.00 were considered as presence in the sample. Proteins expressing >1.5-fold change in abundance with *p* < 0.05 from the duplicate protein extracts were considered as differentially expressed proteins when compared to those of cells grown under normal conditions.

### Quantitative real-time PCR assay of the genes involved in butanol stress response in *B. subtilis* 168

Ten milliliters of the culture grown under the indicated conditions (either with or without butanol treatment at 1.2, 1.4%, or under salt stress using 0.4 M NaCl) were collected for RNA extraction using Nucleospin RNA II kit following the manufacture instruction (Macherey-Nagel, USA). The quantitative real-time RT-PCR (qRT-PCR) was conducted using One-step SYBR PrimeScript RT-PCR kit (Takara Bio Inc., Japan), and by a Lightcycler 1.5 thermocycler (Roche Diagnostics, USA). Primers used in this study are listed in Additional file 5. For normalization of relative gene expression level, a house-keeping gyrase B gene (*gyrB*) was used as an internal control and for calculation. Data were collected from at least three biological replicates.

### HPLC analysis of intracellular glutamate in butanol-treated *B. subtilis* 168

Intracellular content of glutamate was determined using a modified technique of Kuhlmann and Bremer [55]. In brief, twenty milliliters of the culture, either with or without butanol treatment at 1.2, 1.4%, or under salt stress using 0.4 M NaCl, was collected, washed, and lyophilized. Cell pellet dry weight was determined, and extracted with methanol–chloroform–water mixture. The dried solvent extract was dissolved in 60 µl of water and then 1–2 µl was subjected to derivatization with *o*-phthalaldehyde, and analyzed using a reverse phase HPLC following the previously described method [55]. The quantitative analysis was conducted using a standard glutamate.

### Intracellular proline assay in butanol-treated *B. subtilis* 168

The colorimetric assay developed by Bates et al. was used with some modifications [56]. Twenty milliliters of the culture, either with or without butanol treatment at 1.2, 1.4%, or under salt stress using 0.4 M NaCl, was collected and washed twice with SMM. Cell pellet was extracted with 1 ml of 3% 5-sulfosalicylic acid for overnight. Cell debris was removed by centrifugation. One milliliter of the intracellular free amino acid was reacted with 0.5 ml acid ninhydrin solution and 0.5 ml glacial acetic acid. The reaction mixture was incubated in boiling water (100 °C) for 1 h, and then immediately terminated on ice bath. Toluene (2 ml) was added, mixed vigorously, and stored at room temperature for 15 min. The upper layer was used to measure the absorbance at 520 nm against toluene as a blank. Standard proline at 10–200 µM range was used for a quantitative determination.

### Genetic manipulation and gene disruption

Standard genetic manipulation, standard media, and antibiotic concentrations for *B. subtilis* were used according to Kataoka et al. [19]. Disruption of *gltP*, *proB*, and *proHJ* genes was conducted by an antibiotic cassette insertion. The target gene was cloned into pUC119. The antibiotic resistance gene cassette was subcloned and inserted into the target gene for gene disruption. Primers, restriction enzyme sites, and plasmids used in this study are listed in Additional file 5. The plasmid carrying the disrupted gene was then transformed to *B. subtilis*. The homologous recombinant strain was selected in LB medium supplemented with an appropriate antibiotic.

Construction of *gltP* and *proHJ* overexpression plasmids: a *Bacillus*–*E. coli* shuttle vector, pHZK-PX, was used as a template [19] to construct pHK with *gltp* or *proHJ*. The primers, F-phk and R-phk, with multiple cloning sites were designed for PCR amplification of the pHK backbone without P_xyl_GAL region. P_43_ promoter, *gltP*, and *proHJ* were amplified using the indicated primers. Each fragment was restriction digested accordingly. The fragment of *gltP* with P_43_ promoter was then ligated with pHK resulting in pHK-GPOX (HK-GPOX). The fragment of *proHJ* with its own promoter was then ligated with pHK resulting in pHK′-HJOX (HK′-HJOX). The constructed plasmid was subsequently transformed into *B. subtilis* 168 and selected in LB medium supplemented with kanamycin.

All of the mutants were then tested for their genotypes and phenotypes to confirm the expected characteristics (Additional file 4).

### Statistical analysis

All statistical analyses were conducted using GraphPad InStat3 (CA, USA). One way Analysis of variance was done at *p* value of 0.05. Student–Newman–Keuls was used for a multiple comparisons test.

## Additional files

**Additional file 1.** One-dimensional SDS-PAGE for protein separation of *B. subtilis* 168 with and without butanol treatment.

**Additional file 2.** Analysis and identification of polypeptides differentially expressed in *B. subtilis* 168 with butanol stress at 1.2 and 1.4% (vol/vol) in comparison to those of the non-butanol treated cells.

**Additional file 3.** Specific growth rate of the 168 wild-type, the mutants, and the strains with overexpressed gene grown in LB medium in the presence or absence of butanol stress.

**Additional file 4.** The experimental data to verify the mutants and the strains with gene overexpression.

**Additional file 5.** Primers used in this study.

## Abbreviations

*B. subtilis*: *Bacillus subtilis*
LB: Luria–Bertani
SMM: Spizizen minimal medium
SDS-PAGE: sodium dodecyl sulfate polyacrylamide gel electrophoresis
qRT-PCR: quantitative real-time reverse transcription polymerase chain reaction
HPLC: high-performance liquid chromatography
LC: liquid chromatography
MS: mass spectrometry
