# Supplementary material for: The significance of proline and glutamate on butanol chaotropic stress in Bacillus subtilis 168
Source: Biotechnol Biofuels. 2017 May 11;10:122. doi: 10.1186/s13068-017-0811-3 (PMC5425972; doi:10.1186/s13068-017-0811-3)

Additional file 1:

One-dimensional SDS-PAGE for protein separation of *B. subtilis* 168 with and without butanol treatment

The protein profile of *B. subtilis* cells without butanol treatment was referred to as “control”, while those with butanol treatment at 1.2% and 1.4% (vol/vol) for 6 h were referred to as 1.2%, and 1.4%, respectively.

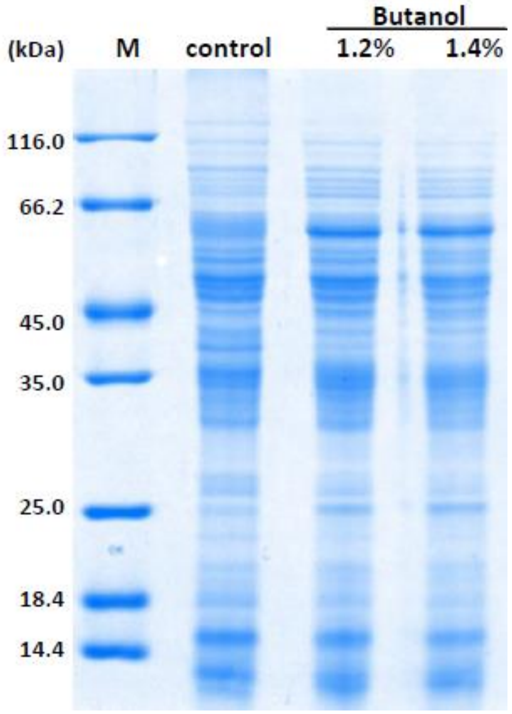

Supplement: Supplementary file 1 — Additional file 1. One-dimensional SDS-PAGE for protein separation of B. subtilis 168 with and without butanol treatment. [file 13068_2017_811_MOESM1_ESM.pdf]
