# Supplementary material for: The significance of proline and glutamate on butanol chaotropic stress in Bacillus subtilis 168
Source: Biotechnol Biofuels. 2017 May 11;10:122. doi: 10.1186/s13068-017-0811-3 (PMC5425972; doi:10.1186/s13068-017-0811-3)
Supplement: Supplementary file 2 — Additional file 2. Analysis and identification of polypeptides differentially expressed in B. subtilis 168 with butanol stress at 1.2 and 1.4% (vol/vol) in comparison to those of the non-butanol treated cells. [file 13068_2017_811_MOESM2_ESM.pdf]

## Additional file 2:

## Analysis and identification of polypeptides differentially expressed in *B. subtilis* 168 with butanol stress at 1.2% and 1.4% (vol/vol) in comparison to those of the non-butanol treated cells

| Protein fold change of the butanol-stressed cells compared to that of the non-treated cells | | | | | | | | |
|---|---|---|---|---|---|---|---|---|
| Down-regulated cut off level | | | | Normal level | Up-regulated cut off level | | | |
| ≤ -2.00 | -1.99 to -1.75 | -1.74 to -1.50 | -1.49 to -1.25 | -1.24 to 1.24 | 1.25 to 1.49 | 1.50 to 1.74 | 1.75 to 1.99 | ≥2.00 |

| Metabolic group (in parenthesis: number of proteins involved) | Protein identification | Gene | Accession number | Biological function and brief information | Protein fold change under butanol stress | |
|---|---|---|---|---|---|---|
| | | | | | 1.2% BtOH | 1.4% BtOH |
| **Protein and amino acid metabolisms (27)** | | | | | | |
| **Protein biosynthesis (22)** | Ribosomal large subunit pseudouridine synthase B | *rluB* | GI:251757325 | rRNA processing | 2.17 | 2.55 |
| | **Ribonuclease R** | ***rnr**** | GI:7674332 | rRNA processing | 1.61 | 1.13 |
| | 50S ribosomal protein L3 | *rplC* | GI:1710556 | rRNA processing | -1.28 | -1.61 |
| | Elongation factor G | *fusA* | GI:239938902 | Translation: It involves in GTP-dependent ribosomal translocation step during translation elongation. | 2.05 | 1.58 |
| | 50S ribosomal protein L1 | *rplA* | GI:239938932 | Translation regulation | -1.12 | -2.04 |
| | Asparagine--tRNA ligase | *asnS* | GI:1711642 | tRNA synthesis | 2.04 | 1.52 |
| | Aspartyl/glutamyl-tRNA(Asn/Gln) amidotransferase subunit B | *gatB* | GI:7674034 | tRNA synthesis | 1.51 | 1.23 |
| | Glutamate--tRNA ligase | *gltX* | GI:135106 | tRNA synthesis | 1.87 | 1.20 |
| | Isoleucine--tRNA ligase | *ileS* | GI:239938843 | tRNA synthesis | 1.52 | 1.01 |
| | Lysine--tRNA ligase | *lysS* | GI:586058 | tRNA synthesis | 1.58 | 1.56 |
| | Methionine--tRNA ligase | *metG* | GI:586060 | tRNA synthesis | 2.11 | 1.32 |
| | Tyrosine--tRNA ligase 1 | *tyrS1* | GI:135192 | tRNA synthesis | 2.44 | 1.64 |
| | Valine--tRNA ligase | *valS* | GI:251757438 | tRNA synthesis | 2.57 | 2.05 |
| | 50S ribosomal protein L4 | *rplD* | GI:1173050 | One of the primary rRNA binding proteins | 1.64 | 1.26 |

| | | | | | | |
|---|---|---|---|---|---|---|
| | 50S ribosomal protein L13 | *rplM* | GI:239938692 | Translation regulation | 1.09 | -1.56 |
| | 50S ribosomal protein L21 | *rplU* | GI:132771 | Translation regulation | 1.63 | 1.26 |
| | 50S ribosomal protein L35 | *rpmI* | GI:2500336 | Ribosomal protein | 1.88 | 1.42 |
| | 30S ribosomal protein S2 | *rpsB* | GI:3123268 | Ribosomal protein | 1.59 | 1.37 |
| | 30S ribosomal protein S4 | *rpsD* | GI:133949 | Ribosomal protein | 1.55 | 1.32 |
| | 30S ribosomal protein S5 | *rpsE* | GI:1173265 | Ribosomal protein | 1.65 | 1.26 |
| | 30S ribosomal protein S7 | *rpsG* | GI:239938934 | Ribosomal protein | 1.22 | 1.63 |
| | 30S ribosomal protein S9 | *rpsI* | GI:2507325 | Ribosomal protein | 1.76 | 1.29 |
| **Amino acid metabolism (5)** | 1-pyrroline-5-carboxylate dehydrogenase 1 | *rocA* | GI:730598 | Arginine metabolism, Glutamate metabolism: It involves in L-proline degradation into L-glutamate; L-glutamate from L-proline. The expression is sigma L-dependent and induced by arginine. | 1.77 | 1.76 |
| | Ornithine aminotransferase | *rocD* | GI:730217 | Arginine metabolism, Proline biosynthesis: It catalyzes the interconversion of ornithine to glutamate semialdehyde. | 2.15 | 1.96 |
| | 1-pyrroline-5-carboxylate dehydrogenase 2 | *putC, ycgN* | GI:239938699 | Glutamate metabolism, Proline metabolism: It involves in L-proline degradation into L-glutamate. | 1.55 | 1.17 |
| | Phosphoserine aminotransferase | *serC* | GI:3041731 | Serine biosynthesis | 1.84 | 1.24 |
| | 3-dehydroquinate dehydratase | *yqhS* | GI:1703004 | Aromatic amino acid biosynthesis | 1.60 | 1.34 |

| **Carbohydrate metabolism** | | | | | | |
|---|---|---|---|---|---|---|
| | Acetoin reductase/2,3-butanediol dehydrogenase | *bdhA* | GI:81815783 | Butanoate metabolism | 1.52 | 1.06 |
| | 6-phosphogluconate dehydrogenase | *gndA* | GI:33518617 | Gluconate utilization, Pentose shunt | 1.56 | 1.11 |
| | Pyruvate carboxylase | *pyc* | GI:46397656 | Gluconeogenesis | 1.69 | 1.53 |
| | Probable L-serine dehydratase | *sdaAA* | GI:6094255 | Gluconeogenesis | 1.68 | 1.60 |
| | Triosephosphate isomerase | *tpiA* | GI:6175067 | Gluconeogenesis, Glycolysis, Pentose shunt | 1.91 | 2.03 |
| | Aerobic glycerol-3-phosphate dehydrogenase | *glpD* | GI:239938636 | Glycerol metabolism | 1.78 | 1.33 |
| | Glyceraldehyde-3-phosphate dehydrogenase 1 | *gapA* | GI:120685 | Glycolysis | 1.55 | 1.01 |
| | Glyceraldehyde-3-phosphate dehydrogenase 2 | *gapB* | GI:3122129 | Glycolysis | 2.02 | 1.64 |
| | Pyruvate dehydrogenase E1 component subunit alpha | *pdhA* | GI:3123238 | Glycolysis | 1.90 | 1.87 |
| | Pyruvate dehydrogenase E1 component subunit beta | *pdhB* | GI:129068 | | 1.51 | 1.26 |
| | ATP-dependent 6-phosphofructokinase | *pfkA* | GI:3122295 | Glycolysis | 1.90 | 1.03 |
| | Pyruvate kinase | *pyk* | GI:3183541 | Glycolysis | 1.42 | 1.57 |
| | Probable fructose-bisphosphate | *fbaA* | GI:543796 | Glycolysis, Sporulation | 1.62 | 1.16 |

| | | | | | | |
|---|---|---|---|---|---|---|
| | aldolase | | | | | |
| | Transketolase | *tkt* | GI:2507484 | Pentose phosphate pathway | 1.61 | 1.20 |
| | Trehalose-6-phosphate hydrolase | *treA* | GI:7404478 | Starch and sucrose metabolism:<br>It involves in trehalose catabolic process | 1.57 | 1.45 |
| | Ribokinase | *rbsK* | GI:3915836 | D-ribose degradation | 1.35 | 1.63 |

| **Stress responses** | | | | | | |
|---|---|---|---|---|---|---|
| | Transcription elongation factor GreA | *greA* | GI:3183527 | Transcription and regulation | 1.82 | 1.43 |
| | **UTP--glucose-1-phosphate uridylyltransferase** | ***gtaB**** | GI:585225 | Carbohydrate metabolism | 1.58 | 1.42 |
| | Alkyl hydroperoxide reductase subunit C | *ahpC* | GI:1703216 | Oxidative stress:<br>It involves in reduction of organic hydroperoxides. | 1.87 | 1.18 |
| | Vegetative catalase | *katA* | GI:239938948 | Oxidative stress:<br>It involves in cell protection from the toxic effects of hydrogen peroxide. | 1.57 | 1.12 |
| | **ATP-dependent Clp protease proteolytic subunit** | ***clpP**** | GI:3287962 | Protein folding | 1.45 | 1.64 |
| | Chaperone protein DnaK | *dnaK* | GI:118703 | Protein folding | 1.72 | 1.62 |
| | 60 kDa chaperonin | *groL* | GI:116230 | Protein folding | 1.88 | 1.98 |
| | 10 kDa chaperonin | *groS* | GI:399235 | Protein folding | 1.75 | 1.42 |
| | Lon protease 1 | *lonA* | GI:585415 | Protein folding | 1.63 | 1.35 |
| | **General stress protein 20U** | ***dps**** | GI:3183517 | Cellular iron ion homeostasis | 1.54 | 1.28 |
| | **Stressosome protein RsbRA** | ***rsbRA**** | GI:1175726 | It acts as a positive regulator of sigma-B activity in response to salt and heat stress. | 1.85 | 1.55 |
| | **Putative cysteine protease YraA** | ***yraA**** | GI:37999917 | It functions in the protection against aldehyde-stress, possibly by degrading damaged proteins. | 1.76 | 1.81 |

| **Tricarboxylic acid cycle and energy metabolism (12)** | | | | | | |
|---|---|---|---|---|---|---|
| **Tricarboxylic acid cycle (8)** | Aconitate hydratase A | *citB* | GI:2506131 | Catabolism of short chain fatty acids | 1.28 | 1.51 |
| | Malate dehydrogenase | *mdh* | GI:1708969 | Reversible oxidation of malate to oxaloacetate. | 1.50 | 1.29 |
| | 2-oxoglutarate dehydrogenase E1 | *odhA* | GI:254763409 | Conversion of 2-oxoglutarate to succinyl-CoA and CO2. | 1.73 | 1.28 |
| | 2-oxoglutarate dehydrogenase complex E2 | *odhB* | GI:251757302 | The 2-oxoglutarate dehydrogenase complex catalyzes the overall conversion of 2-oxoglutarate to succinyl-CoA and CO2. | 2.10 | 1.41 |
| | Succinate dehydrogenase flavoprotein subunit | *sdhA* | GI:251757490 | Fumarate synthesis from succinate | 1.68 | 1.09 |
| | Succinate dehydrogenase iron-sulfur subunit | *sdhB* | GI:118613 | Fumarate synthesis from succinate | 1.51 | 1.23 |
| | Succinyl-CoA ligase [ADP-forming] subunit beta | *sucC* | GI:3183562 | Succinate conversion | 1.43 | 1.59 |
| | Succinyl-CoA ligase [ADP-forming] subunit alpha | *sucD* | GI:3915596 | Succinate conversion | 1.69 | 1.04 |
| **Energy** | ATP synthase subunit beta | *atpD* | GI:584808 | ATP synthesis | 2.27 | 1.75 |
| | ATP synthase subunit *b* | *atpF* | GI:584815 | ATP synthesis | 1.51 | 1.18 |

| | | | | | | |
|---|---|---|---|---|---|---|
| **metabolism (4)** | | | | | | |
| | ATP synthase gamma chain | *atpG* | GI:226693512 | ATP synthesis | 1.50 | 1.37 |
| | Cytochrome *bd* ubiquinol oxidase subunit 1 | *cydA* | GI:2829796 | Electron transport, Transport | 1.82 | 1.71 |
| **Genetic information processing (10)** | | | | | | |
| **DNA replication (3)** | Inosine-5'-monophosphate dehydrogenase | *guaB* | GI:34395945 | GMP biosynthesis, Purine biosynthesis | 1.56 | 1.86 |
| | DNA topoisomerase 4 subunit A | *parC* | GI:239938679 | Chromosome segregation | 1.60 | 1.06 |
| | ATP-dependent helicase PriA | *priA* | GI:3183549 | DNA replication forks. | 1.90 | 1.50 |
| **Transcription and regulation (7)** | DNA-directed RNA polymerase subunit beta | *rpoB* | GI:239938700 | Transcription | 1.69 | 1.37 |
| | DNA-directed RNA polymerase subunit beta' | *rpoC* | GI:239938935 | Transcription | 1.63 | 1.28 |
| | **Negative regulator of genetic competence ClpC/MecB** | ***clpC**** | GI:586900 | Competence gene repressor | 1.70 | 1.22 |
| | GTP-sensing transcriptional pleiotropic repressor CodY | *codY* | GI:729175 | DNA Regulation | 2.24 | 1.45 |
| | Sporulation-control protein spo0M | *spo0M* | GI:239938711 | Sporulation | 1.62 | 1.61 |
| | Transcriptional regulatory protein ResD | *resD* | GI:251757323 | Transcription regulation, Two-component regulatory system | 1.50 | 1.28 |
| | Uncharacterized transcriptional regulatory protein YcbL | *ycbL* | GI:238054356 | Transcription regulation, Two-component regulatory system | 1.57 | 1.07 |
| **Biosynthesis of antibiotics and vitamins (7)** | | | | | | |
| **Antibiotic biosynthesis (4)** | 3-oxo-glucose-6-phosphate:glutamate aminotransferase | *ntdA* | GI:71648722 | It involves in the biosynthesis of kanosamine | 1.75 | 1.23 |
| | Surfactin synthase subunit 1 | *srfAA* | GI:239938937 | Surfactin synthase | 1.77 | 1.41 |
| | Surfactin synthase subunit 2 | *srfAB* | GI:239938827 | Surfactin synthase | 1.50 | 1.20 |
| | Polyketide synthase PksN | *pksN* | GI:254782318 | Bacillaene biosynthesis | 1.77 | 1.65 |
| **Vitamin biosynthesis (3)** | 8-amino-7-oxononanoate synthase 1 | *kbl* | GI:6685543 | Biotin biosynthesis | 1.82 | 1.67 |
| | 1,4-dihydroxy-2-naphthoyl-CoA synthase | *menB* | GI:20141516 | Menaquinone biosynthesis | 1.91 | 1.37 |
| | 7-cyano-7-deazaguanine synthase | *queC* | GI:81341924 | Queuosine biosynthesis | 1.59 | 1.36 |
| **Lipid metabolism and cell division (4)** | | | | | | |
| **Lipid metabolism (2)** | Enoyl-[acyl-carrier-protein] reductase [NADH] FabI | *fabI* | GI:7531269 | Fatty acid biosynthesis | 1.79 | 1.40 |
| | 3-oxoacyl-[acyl-carrier-protein] | *fabG* | GI:3915689 | Fatty acid biosynthesis | 1.95 | 1.19 |

| | | | | | | |
|---|---|---|---|---|---|---|
| | reductase FabG | | | | | |
| **Cell division (2)** | UDP-N-acetylglucosamine 1-carboxyvinyltransferase 1 | *murAA* | GI:7674128 | Cell shape, Cell wall biogenesis/degradation, Peptidoglycan synthesis | 1.58 | 1.64 |
| | Teichuronic acid biosynthesis protein TuaB | *tuaB* | GI:46577302 | Cell wall biogenesis/degradation, Stress response | 1.63 | 1.10 |

| **Others (20)** | | | | | | |
|---|---|---|---|---|---|---|
| | Acetyl-coenzyme A synthetase | *acsA* | GI:728788 | Carbon metabolism | 3.75 | 3.77 |
| | Oligopeptide-binding protein OppA (Stage 0 sporulation protein KA) | *oppA* | GI:129179 | Competence, Peptide transport, Protein transport, Sporulation, Transport | 1.67 | 1.58 |
| | Uncharacterized protein YuaB | *yuaB* | GI:3183498 | Membrane architecture | 1.50 | -1.14 |
| | Nitrate reductase alpha chain | *narG* | GI:254763315 | Electron transport, Nitrate assimilation, Transpor | 1.90 | 1.72 |
| | Vegetative protein 296 | *sufC* | GI:3183561 | Transport | 1.46 | 1.64 |
| | Putative aldehyde dehydrogenase DhaS | *dhaS* | GI:81669150 | Cellular aldehyde metabolic process | 1.89 | 1.47 |
| | Protease HtpX homolog | *htpX* | GI:6016277 | Metalloendopeptidase activity | 1.59 | 1.22 |
| | NAD kinase 2 | *nadK2* | GI:24418604 | The biosynthesis of NADP. | 1.69 | 1.33 |
| | FeS cluster assembly protein SufB | *sufB* | GI:81342165 | Iron uptake from extracellular iron chelators under iron limitation. | 1.97 | 2.09 |
| | FeS cluster assembly protein SufD | *sufD* | GI:81342166 | | 1.55 | 1.27 |
| | Uncharacterized sugar epimerase YhfK | *yhfK* | GI:81341012 | unknown | 1.64 | 1.36 |
| | Putative phosphoesterase YjcG | *yjcG* | GI:81341898 | unknown | 1.54 | 1.28 |
| | Uncharacterized protein YjoA | *yjoA* | GI:45477244 | unknown | 1.45 | -2.17 |
| | Uncharacterized protein YpgR | *ypgR* | GI:1730911 | unknown | 1.89 | 1.46 |
| | Putative aminopeptidase YsdC | *ysdC* | GI:81637725 | unknown | 2.59 | 2.02 |
| | NADH dehydrogenase-like protein YutJ | *yutJ* | GI:254807494 | unknown | 1.60 | 1.24 |
| | Uncharacterized protein YvdC | *yvdC* | GI:81342194 | unknown | 1.55 | 1.61 |
| | Uncharacterized protein YvpB | *yvpB* | GI:81637596 | unknown | 1.42 | 1.56 |
| | Uncharacterized ABC transporter permease YvrN | *yvrN* | GI:239938819 | unknown | 1.67 | -1.33 |
| | Uncharacterized protein YvyG | *yvyG* | GI:732323 | unknown | 1.58 | 1.34 |

*Note:* Amino acid sequences were searched against *B. subtilis* subsp. subtilis 168 protein database. Peptides that were unique in the protein database with 100% identity and 100% query coverage were considered as a true match. Gene, protein name and functions were acquired from true match peptides and grouped according to Uniport database.

*Gene members in SigB-dependent regulon or SigB positive regulator [31].
