# Supplementary material for: The significance of proline and glutamate on butanol chaotropic stress in Bacillus subtilis 168
Source: Biotechnol Biofuels. 2017 May 11;10:122. doi: 10.1186/s13068-017-0811-3 (PMC5425972; doi:10.1186/s13068-017-0811-3)
Supplement: Supplementary file 3 — Additional file 3. Specific growth rate of the 168 wild-type, the mutants, and the strains with overexpressed gene grown in LB medium in the presence or absence of butanol stress. [file 13068_2017_811_MOESM3_ESM.pdf]

Additional file 3:

Specific growth rate of the 168 wild-type, the mutants, and the strains with overexpressed gene grown in LB medium in the presence or absence of butanol stress.

**Strains (Description)**	**Specific growth rate under various conditions**			
	**Non-stress control**	**1.4% BtOH**	**1.6% BtOH**	**1.8% BtOH**
168 (Wildtype strain)	0.263 ± 0.007	0.147 ± 0.002	ND	ND
146 (Δ*proBA* mutant)	0.277 ± 0.003	0.015 ± 0.005	ND	ND
B934 (Δ*proB* mutant)	0.267 ± 0.002	0.069 ± 0.002	ND	ND
BH901 (Δ*proB* Δ*proHJ* mutant)	0.249 ± 0.006	0.014 ± 0.005	ND	ND
H972 (Δ*proHJ* mutant)	0.230 ± 0.006	0.118 ± 0.011	ND	ND
GP16 (Δ*gltP* mutant)	0.278 ± 0.007	0.085 ± 0.019	ND	ND
HK (Strain harboring an empty vector control)	0.123 ± 0.003	ND	0.009 ± 0.003	0
HK-GPOX (Strain with *gltP* overexpression)	0.122 ± 0.002	ND	0.044 ± 0.002	0.019 ± 0.006
HK'-HJOX (Strain with *proHJ* overexpression)	0.100 ± 0.002	ND	0.036 ± 0.001	0.014 ± 0.003

Note: ND: Not determined. Data are means of the results ± S.D. from at least three individual experiments.
