# Supplementary material for: The significance of proline and glutamate on butanol chaotropic stress in Bacillus subtilis 168
Source: Biotechnol Biofuels. 2017 May 11;10:122. doi: 10.1186/s13068-017-0811-3 (PMC5425972; doi:10.1186/s13068-017-0811-3)
Supplement: Supplementary file 4 — Additional file 4. The experimental data to verify the mutants and the strains with gene overexpression. [file 13068_2017_811_MOESM4_ESM.pdf]

Additional file 4:

The experimental data to verify the mutants and the strains with gene overexpression

5.1 Verification of the mutants by genomic PCR.

5.1.1 Verification of the mutant strain GP16 (*gltP* mutant)

The *gltP* mutant [168Δ(*gltP*::*cam*^{r})] was constructed by insertion of chloramphenicol resistance gene (*cam*^{r} or *cat*) in *gltP* wild-type. The upstream region of *gltP* (UgltP) was amplified by F-5U*gltP*-*Sal*I and R-5U*gltP*-*Kpn*I and the downstream region of *gltP* (DgltP) was amplified by F-3D*gltP*-*SacI* and R-3D*gltP*-*Eco*RI. The *cat* gene was amplified by F-*cat*-*Kpn*I and R-*cat*-*Sac*I. All three PCR products (UgltP, DgltP, and *cat* gene) were digested by the indicated restriction enzymes and cloned into pUC119 as shown in following figure.

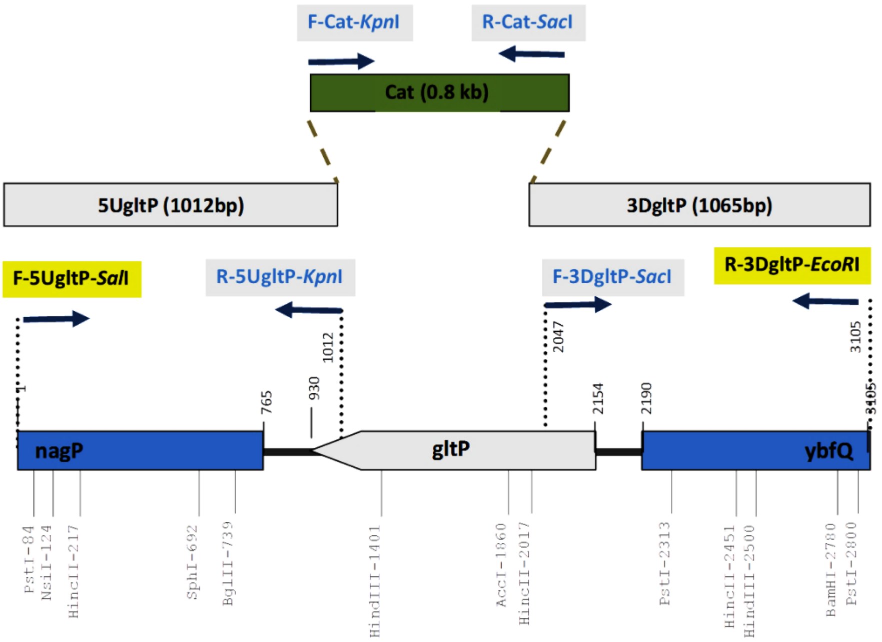

The constructed vector was transformed into *B. subtilis* 168 (WT). The transformants were selected as chloramphenicol resistance clones. The genomic of GP16 (Δ*gltP*::*cam*^{r}) strain was verified by genomic PCR. The pair of primer F-5U*gltP*-*Sal*I and R-3D*gltP*-*Eco*RI (highlighted in yellow) was used for PCR amplification resulting in a 2.8-kb PCR product of mutants and a 3.1-kb PCR product of WT as shown in the following figure. The clone #GP16 was selected for further study.

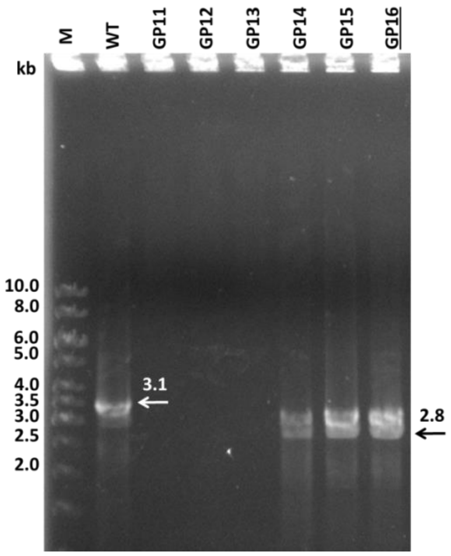

The genomic PCR verification of the *gltP* mutants. M: GeneRuler 1 kb DNA Ladder (Thermo Scientific); WT: *B. subtilis* 168; GP11-GP16: the tested clones

5.1.2 Verification of the mutant strain B934 (*proB* mutant)

The *proB* mutant 168Δ(*proB*::*spc*^{r}) was constructed by insertion of spectinomycin resistance gene (*spc*^{r} or *spc*) in *proB* wild-type. The covered region of *proB* was amplified by F-53*proB* and R-53*proB*-*Sph*I. The *spc* gene was amplified by F-*spc*-*Sac*II and R-*spc*-*Sac*II resulting a 1.05-kb PCR product. The wild-type *proB* gene was cloned into pUC119 at *Eco*RI-*Sph*I site, and then the *spc* gene was flanked into the vector at *Sac*II sites. The construction procedure is shown in the following figure.

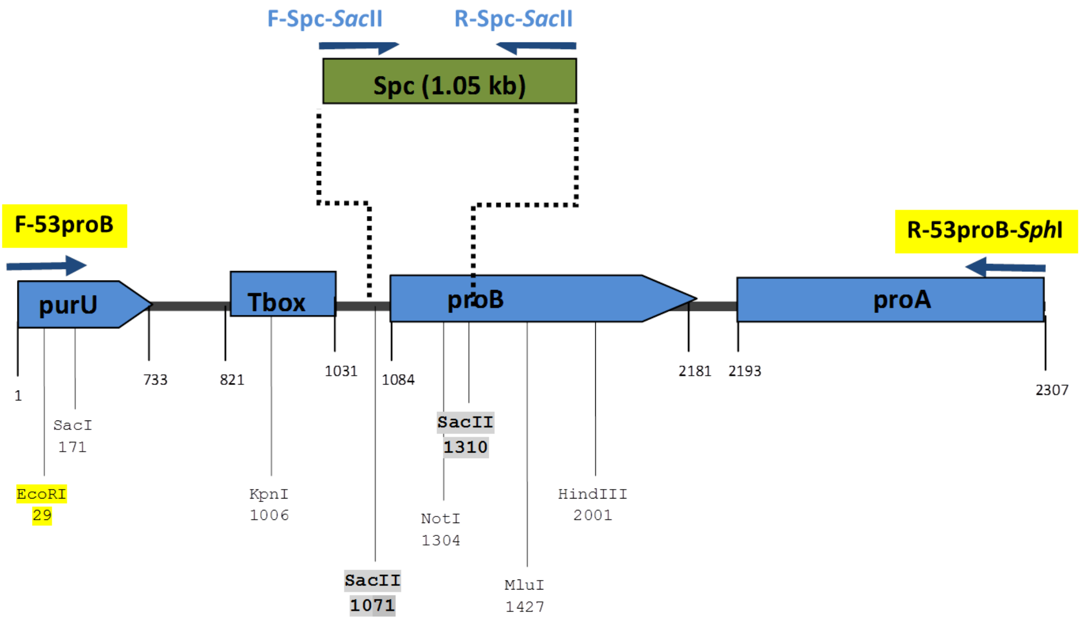

The constructed vector was transformed into *B. subtilis* 168 (WT). The transformants were selected as spectinomycin resistance clones. The mutant strain B934 (Δ*proB*::*spc*^{r}) strain was verified by genomic PCR. The pair of F-53*proB* and R-53*proB*-*Sph*I (highlighted in yellow) was used for PCR amplification resulting in a 2.8-kb PCR product of mutants and

a 2.3-kb PCR product of WT as shown in the following figure. The mutant B934 was used for further experiment, and for the construction of the mutant strain BH901.

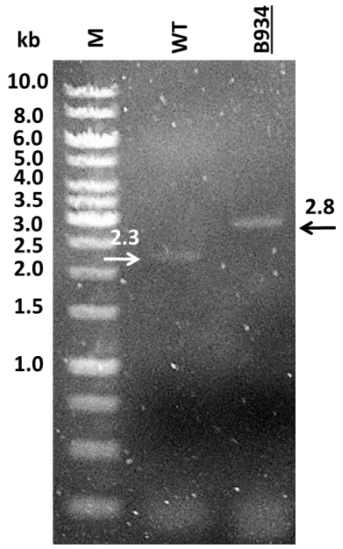

The genomic PCR verification of *proB* mutants. M: GeneRuler 1 kb DNA Ladder (Thermo Scientific); WT: *B. subtilis* 168; B934: 168Δ*proB*::*spc*^{r}

5.1.3 Verification of the mutant strain BH901 (*proHJ* mutant)

The *proHJ* mutant 168Δ(*proB*::*spc*^{r}) Δ(*proHJ*::*cam*^{r}) or B934Δ(*proHJ*::*cam*^{r}) were constructed by insertion of chloramphenicol resistance gene (*cam*^{r} or *cam*) in *proHJ* wild-type. The covered region of *proHJ* was amplified by F-53*proHJ*-*Bam*HI and R-53*proHJ*-*Kpn*I. The *cat* gene was amplified by F-*cat*-*Xho*I and R-*cat*-*Bgl*II resulting a 0.8-kb PCR product. The wild-type *proHJ* gene was cloned into pUC119 at *Bam*HI-*Kpn*I site. Then the *cat* gene was flanked into the vector at *Xho*I-*Bgl*II site. The construction procedure is shown in the following figure

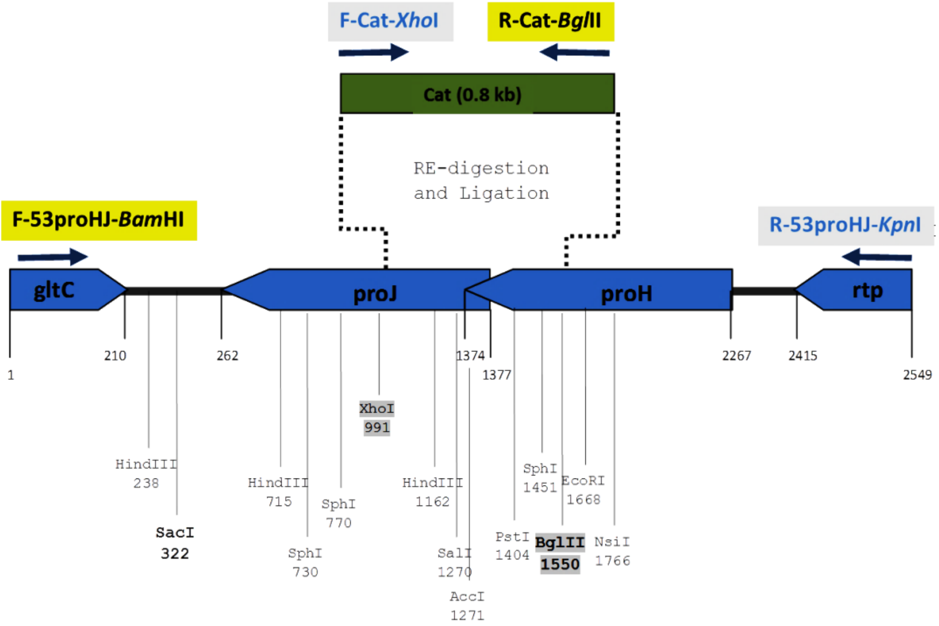

The constructed vector was transformed into *B. subtilis* 168 (WT) and the mutant strain B934. The transformants were then selected as chloramphenicol resistance clones. BH901 mutant strains (Δ*proB*::*spc*^{r}, Δ*proHJ::cam*^{r}) and H972 (Δ*proHJ::cam*^{r}) were verified by genomic PCR. The pair of F-53*proHJ*-*Bam*HI and R-*cat*-*Bgl*II (highlighted in yellow) was used for PCR amplification resulting in a 1.8-kb PCR product as shown in the following figure.

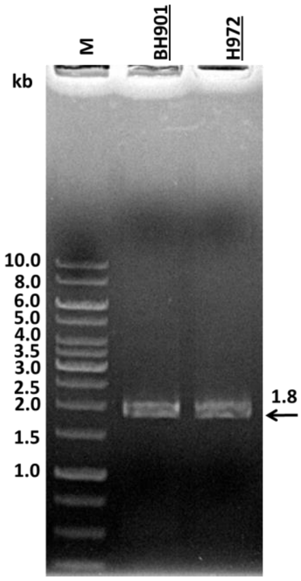

The genomic PCR verification of the *proHJ* mutants. M: GeneRuler 1 kb DNA Ladder (Thermo Scientific); WT: *B. subtilis* 168; BH901: Δ*proB*::*spc*^{r}, Δ*proHJ::cam*^{r}; H972: Δ*proHJ::cam*^{r}.

5.2 Verification of the strains with gene overexpression (HK-GOX and HK'-HJOX) using qRT-PCR.

The strains with the overexpressed gene (HK-GOX: a recombinant strain with an overexpressed GltP under P43 promoter; and HK'-HJOX: a recombinant strain with an overexpressed ProHJ under its original promoter) were verified using qRT-PCR of the gene target. The strains were cultivated in LB medium to early-exponential phase, then further incubated for 1 h, and extracted the RNA. qRT-PCR analysis was carried out using the specific primers indicated in Additional file 3. The level of gene-expression was then compared to that of the HK strain control (168 carrying pHK empty-vector), and expressed as expression fold change (as shown below). The results are mean of two independent biological replicates.

**Strain**	**A target gene**	**Expression fold change**
HK-GPOX	*gltP*	170.1 ± 39.8
HK'-HJOX	*proH*	371.7 ± 23.9
