# Supplementary material for: The significance of proline and glutamate on butanol chaotropic stress in Bacillus subtilis 168
Source: Biotechnol Biofuels. 2017 May 11;10:122. doi: 10.1186/s13068-017-0811-3 (PMC5425972; doi:10.1186/s13068-017-0811-3)
Supplement: Supplementary file 5 — Additional file 5. Primers used in this study. [file 13068_2017_811_MOESM5_ESM.pdf]

Additional file 5:

Primers used in this study

Purpose	Target gene	Primer	Sequence (5’→3’)*	Gene source
Δ*gltP*	5U*gltP*	F-5UgltP-*Sal*I	ATGCGTCGACGCCCGGCCGTGAGACAGATG	gDNA**
		R-5UgltP-*Kpn*I	ATGCGGTACCTCCGACAGGCCTCAGTGCCA	gDNA**
	cat	F-Cat-*Kpn*I	ATGCGGTACCACAAACGAAAATTGGATAAAGTG	pHT01
		R-Cat-*Sac*I	ATGCGAGCTCTTATAAAAGCCAGTCATTAGGCCT	pHT01
	3D*gltP*	F-3DgltP-*Sac*I	ATGCGAGCTCAACAGGCGTGAACGTACCGGG	gDNA**
		R-3DgltP-*EcoR*I	ATGCGAATTCCCAGAGGCGTTTAGAGAGCAGCA	gDNA**
Δ*proB*	53*proB*	F-53proB	ATGCGAATTCTGAAGGCGGCCGGTTCTTCC	gDNA**
		R-53proB-*Sph*I	ATGCTCTAGAGCATGCTGCGGAGTCCGTTTGCAATGAGG	gDNA**
	spc	F-Spc-*Sac*II	ATGCGCCCGGAACGAGGTGAAATCATGAGC	pIC333
		R-Spc-*Sac*II	ATGCGCCCGGTAAATTAAAGTAATAAAGCGTTCTCTAATTTC	pIC333
Δ*proHJ*	53*proHJ*	F-53proHJ-*Bam*HI	ATGCGGATCCGTGTCCGCAGGAATGGGCG	gDNA**
		R-53proHJ-*Kpn*I	ATGCGGTACCGGGGCTAAGCTCCAGGAAGTCG	gDNA**
	cat	F-Cat-*Xho*I	ATGCCTCGAGACAAACGAAAATTGGATAAAGTG	pHT01
		R-Cat-*Bgl*II	ATGCAGATCTTTATAAAAGCCAGTCATTAGGCCT	pHT01
pHK	pHK backbone	F-phk	ATCGGCCGCTAGCGCATGCCTCGAGGTACCGCATCAGAGGGGAATTCCTG<br>*Eag*I *Nhe*I *Sph*I *Xho*I *Kpn*I *Eco*RI	pHZK-PX
		R-phk	ATCGGCCGCGGCCGCCCGGGATCCGTCGACTCTAGAAGCTTGGGCAAAGC<br>*Eag*I *Not*I *Sma*I *Bam*HI *Sal*I *Xba*I *Hind*III	pHZK-PX
gltP and *proHJ* over-expression	P_{43}	F-p43	ACTGGGATCC*AGATCT*ACTGACAAACATCACCCTCT<br>*Bam*HI *Sph*I	gDNA**
		R-p43	ACTGGTCGACTGGTACCGCTATCACTTTAT<br>*Sal*I	gDNA**
	gltP	F-gltPOX	ACTGGCGGCCGC*aaaggagg*aaggatcaATGAAAAAATTAATCGCGTTTC<br>*Not*I RBS Spacer	gDNA**
		R-gltPOX	AGTCCTCGAGTCTAGACACCTCGTATTGAGGTGTTC<br>*Xho*I *Xba*I	gDNA**
	proHJ	F-53proHJ-*Bam*HI	ATGCGGATCCGTGTCCGCAGGAATGGGCG	gDNA**
		R-53proHJ-*Kpn*I	ATGCTGTAGAGGTACCGGGGCTAAGCTCCAGGAAGTCG	gDNA**
qRT-PCR	*gyrB*	qF-gyrB168	AAGCTGGGCAACTCAGAAGCACGG	
		qR-gyrB168	AGCCATTCTTGCTCTTGCCGCC	
	opuE	qF-opuE168	ATGAACGAATGGGGCGCGCTTG	
		qR-opuE168	ACCGGTTGATTTTGCCAGGCCG	
	proA	qF-proA168	AGACAGGGAACGCGGTTGTG	
		qR-proA168	TGCACAGCGTGAATCGGAAGCG	
	proI	qF-proI168	AACGTACAGCGTGCGTCCATGC	
		qR-proI168	ATGCTTTCCGCCGCGTCTTTCG	
	proH	qF-proH168	AAGCATTGCTCGGATGCATGGGC	
		qR-proH168	ATAAAAATACGCGGGTCCGCTGCC	
	gltP	qF-gltP168	TTTCGGGATGGCACTGAGGCCTG	
		qR-gltP168	TTCCGCTTCCTGCGGCTCCAATG	
	gltA	qF-gltA168	TACTTTGCAATGGGCGTGCCGCAG	
		qR-gltA168	TCCGATGCGTTCTTCATCAGCCGC	
	gabT	qF-gabT168	ATCGGGCGTGCGGAAATGCTTG	
		qR-gabT168	TATCCAAGACTGCCAAAGCCGCCG	
	gudB	qF-gudB168	AAAGGTGCGCGTGTCGTTGTCC	
		qR-gudB168	AGGCCTTCCGGATCATAAAGTCCGC	
	glnA	qF-glnA168	TCATGGTCAAAGCGCTGGGC	
		qR-glnA168	CCAAGGATGTACTTGCGTGCGGAAC	
	putP	qF-putP168	TCTTTGGGTCCGGCAGTAAC	
		qR-putP168	AGTCCGATGACGATCCAAGC	
	rocD	qF-rocD168	ATGCAAGCGGGTTTCCACCA	
		qR-rocD168	ATCTCTTGCATTGCGGCGGA	

*Introducing restriction enzyme site was bolded and underlined.

**gDNA represents genomic DNA of *B. subtilis* 168
